# Using RNA-Seq to Investigate Immune-Metabolism Features in Immunocompromised Patients With Sepsis

**DOI:** 10.3389/fmed.2021.747263

**Published:** 2021-12-17

**Authors:** Po-Liang Cheng, Hsin-Hua Chen, Yu-Han Jiang, Tzu-Hung Hsiao, Chen-Yu Wang, Chieh-Liang Wu, Tai-Ming Ko, Wen-Cheng Chao

**Affiliations:** ^1^Department of Medical Research, Taichung Veterans General Hospital, Taichung, Taiwan; ^2^Precision Medicine Center, Taichung Veterans General Hospital, Taichung, Taiwan; ^3^Division of General Internal Medicine, Department of Internal Medicine, Taichung Veterans General Hospital, Taichung, Taiwan; ^4^Big Data Center, Chung Hsing University, Taichung, Taiwan; ^5^Department of Industrial Engineering and Enterprise Information, Tunghai University, Taichung, Taiwan; ^6^Rong Hsing Research Centre for Translational Medicine, Institute of Biomedical Science, Chung Hsing University, Taichung, Taiwan; ^7^Department of Public Health, Fu Jen Catholic University, New Taipei City, Taiwan; ^8^Institute of Genomics and Bioinformatics, National Chung Hsing University, Taichung, Taiwan; ^9^Department of Critical Care Medicine, Taichung Veterans General Hospital, Taichung, Taiwan; ^10^Department of Nursing, Hung Kuang University, Taichung, Taiwan; ^11^Department of Computer Science, Tunghai University, Taichung, Taiwan; ^12^Department of Automatic Control Engineering, Feng Chia University, Taichung, Taiwan; ^13^Department of Biological Science and Technology, National Chiao Tung University, Hsinchu, Taiwan; ^14^Department of Biological Science and Technology, National Yang Ming Chiao Tung University, Hsinchu, Taiwan; ^15^Institute of Bioinformatics and Systems Biology, National Chiao Tung University, Hsinchu, Taiwan; ^16^Institute of Biomedical Sciences, Academia Sinica, Taipei, Taiwan

**Keywords:** RNA-Seq, sepsis, immunocompromised, immune, metabolism, pathway analyses

## Abstract

**Objective:** Sepsis is life threatening and leads to complex inflammation in patients with immunocompromised conditions, such as cancer, and receiving immunosuppressants for autoimmune diseases and organ transplant recipients. Increasing evidence has shown that RNA-Sequencing (RNA-Seq) can be used to define subendotype in patients with sepsis; therefore, we aim to use RNA-Seq to identify transcriptomic features among immunocompromised patients with sepsis.

**Methods:** We enrolled patients who were admitted to medical intensive care units (ICUs) for sepsis at a tertiary referral centre in central Taiwan. Whole blood on day-1 and day-8 was obtained for RNA-Seq. We used Gene Set Enrichment Analysis (GSEA) to identify the enriched pathway of day-8/day-1 differentially expressed genes and MiXCR to determine the diversity of T cell repertoire.

**Results:** A total of 18 immunocompromised subjects with sepsis and 18 sequential organ failure assessment (SOFA) score-matched immunocompetent control subjects were enrolled. The ventilator-day, ICU-stay, and hospital-day were similar between the two groups, whereas the hospital mortality was higher in immunocompromised patients than those in immunocompetent patients (50.0 vs. 5.6%, *p* < 0.01). We found that the top day-8/day-1 upregulated genes in the immunocompetent group were mainly innate immunity and inflammation relevant genes, namely, *PRSS33, HDC, ALOX15, FCER1A*, and *OLR1*, whereas a blunted day-8/day-1 dynamic transcriptome was found among immunocompromised patients with septic. Functional pathway analyses of day-8/day-1 differentially expressed genes identified the upregulated functional biogenesis and T cell-associated pathways in immunocompetent patients recovered from sepsis, whereas merely downregulated metabolism-associated pathways were found in immunocompromised patients with septic. Moreover, we used MiXCR to identify a higher diversity of T cell receptor (TCR) in immunocompetent patients both on day-1 and on day-8 than those in immunocompromised patients.

**Conclusions:** Using RNA-Seq, we found compromised T cell function, altered metabolic signalling, and decreased T cell diversity among immunocompromised patients with septic, and more mechanistic studies are warranted to elucidate the underlying mechanism.

## Background

Sepsis, characterised by a dysregulated host-pathogen inflammation, is a leading cause of death worldwide, and approximately 5.3 million people died from sepsis annually (1). There is a global increase of patients with distinct immunocompromised conditions due to the advance of cancer treatment, increasing biologics, and immunosuppressants for autoimmune diseases and growing organ transplant recipients (2), and it has been estimated that patients with immunocompromised conditions account for approximately 35% of all intensive care unit (ICU) admissions (3). Therefore, there is a crucial need to explore the alternation of immunological responses in sepsis among patients with immunocompromised conditions.

Immunoparalysis, characterised by not only immunologic but also by metabolic dysregulation after sepsis, has been increasingly recognised and attributed to be one of the key biological bases of prolonged impact on long-term mortality in patients with sepsis (4, 5). Recent advances in sequencing technology, such as RNA-Sequencing (RNA-Seq), immune repertoire sequencing, and single-cell RNA-Seq (sc-RNA-Seq), allow for addressing immunological and metabolic features in patients with sepsis and coronavirus disease 2019 (COVID-19) infection (6–8). Several studies have used MicroArray and RNA-Seq to identify the transcriptomic signature, so-called subendotype, in patients with sepsis, and the currently identified sepsis-associated subendotypes included early improvement of organ dysfunction after sepsis and the responsiveness of steroids in patients with a septic shock (6, 9, 10). Additionally, analytic tools, namely, MiXCR, have been recently developed to investigate the diversity of T cell receptor (TCR) using bulk RNA-Seq data (11, 12). Despite increasing studies to explore transcriptomic features of sepsis, the transcriptomic signature of sepsis among immunocompromised patients remains unclear. In the present study, we used RNA-Seq to identify distinct transcriptomic features and MiXCR to explore the diversity of TCR between immunocompromised and severity-matched immunocompetent patients with sepsis.

## Materials and Methods

### Ethical Approval

This study was approved by the Institutional Review Board approval of the Taichung Veterans General Hospital (CE20069B). Informed consent was obtained from all participants prior to the enrolment in the study and collection of blood samples.

### Definition of Patients With Immunocompromised Conditions

Patients were defined as immunocompromised if they had at least one of the following conditions, such as patients receiving immunosuppressive therapy due to autoimmune disease, organ transplant recipient, and patients with active malignant disease consisting of active haematological disease, or solid tumour under therapy (13).

### Enrolment of Subjects

We enrolled 18 immunocompromised patients and 18 severity-matched (day-1 sequential organ failure assessment (SOFA) score ± 1) immunocompetent patients who were admitted to the medical ICUs between March 2020 and February 2021 at Taichung Veterans General Hospital, a tertiary referral centre in central Taiwan, for sepsis and extracted blood samples on day-1 and day-8 after the ICU admission. Patients who did not survive until day-8 were not included for analyses; therefore, each enrolled patient for analyses had paired day-1 and day-8 samples in this study. In the present study, we used PAXgene Blood RNA Tube to collect blood samples from enrolled subjects. Clinical variables, such as comorbidities, severity scores (day-1 SOFA score, day-3 SOFA score, day-7 SOFA score, and Acute Physiology and Chronic Health Evaluation (APACHE) II score), laboratory data, and outcomes (ventilator-day, ICU-stay, hospital-stay, and overall mortality) were recorded.

### RNA Preparation and Sequencing

We used PAXgene Blood RNA Kit to extract RNA, and the average RNA integrity number (RIN) was 8.31 ± 0.58 in the present study. The library was constructed in accordance with the manufacturer's instruction, and 800–1,000 ng fragmented RNA was used for further experiments. The RNA-Seq was conducted on the NovaSeq platform (Illumina, San Diego, CA, USA), with at least 50–60 million 150-bp pair-end reads per sample.

### Bioinformatics Analyses

The quality of sequencing was good, and Phred scores 30 was applied, and sequence reads were mapped to the human reference genome GRCh38 by HISAT2 (14). Read counts were calculated by featureCounts (15), and the differentially expressed genes were identified by R package DEseq2 (16). The average mapped rate and read counts were 82.3 ± 7.8% and 104.0 ± 43.6 million reads, respectively. Gene Set Enrichment Analysis (GSEA) was used for functional annotation of the whole differentially expressed genes (17), and the visualised enrichment map was performed by using Cytoscape 3.8.2 (18). In brief, we used an enrichment map, which organise gene sets into a similarity network, to visualise GSEA results, and the node, link, and node colour represent gene-set, the overlap of member gene, and enrichment score, respectively (19).

### Diversity of TCR Analyses

Raw sequences from RNAseq were conducted into MiXCR v3.0.13 (11, 12) to quantitate the clonotypes of patients with sepsis. After obtaining the quantitated clonotypes, VDJTools v 1.2.1 (20) was used to calculate the sample diversity and counts of complementarity-determining region-3 (CDR3).

### Statistical Analyses

Continuous variables were presented as median (interquartile range), and data for categorical variables were shown as numbers (percentages). The differences between the immunocompromised and immunocompetent groups were analysed by the Mann-Whitney U test for continuous variables and Fisher's exact test for categorical variables. Statistical analyses were two-sided, and the level of significance was set at 0.05 for clinical data. Data analyses were conducted using R version 4.0.2.

## Results

### Patient Characteristics

A total of 36 patients with sepsis were enrolled, consisting of 18 immunocompromised patients and 18 day-1 SOFA score-matched immunocompetent control subjects. Of the 18 immunocompromised patients, the number of subjects with solid tumour receiving therapy, haematological cancer, those with autoimmune disease underwent immunosuppressants, and renal transplant recipients were 3, 6, 7, and 2, respectively. The comorbidities and laboratory data were comparable between the two patient groups. With respect to the outcome, the ventilator-day, ICU-stay, and hospital-day were similar between the two groups, whereas the hospital mortality was much higher in immunocompromised patients compared to those in immunocompetent patients (50.0 vs. 5.6%, *p* < 0.01; Table 1; refer to Supplemental Table 1 for details with regards to pathogens and infection sites).

**Table 1:** Characteristics of the enrolled immunocompromised and severity-matched immunocompetent critically ill patients with septic.

	**Immunocompromised**	**Immunocompetent**	***p*-value**
	***n =* 18**	***n =* 18**	
**Demographic data**			
Age (years)	58.0 (50.3–68.3)	71.0 (57.5–81.5)	0.06
Sex (female)	9 (50.0%)	6 (33.3%)	0.50
**Immunocompromised factors**			NA
Solid tumour receiving therapy	4 (22.2%)	NA	
Haematological cancer	5 (27.8%)	NA	
Autoimmune disease	7 (38.9%)	NA	
Renal transplant recipient	2 (11.1%)	NA	
**Severity scores**			
APACHE II score	28.0 (23.0–31.3)	26.0 (22.5–29.5)	0.32
SOFA score, day-1	11.0 (8.5–15.0)	12.0 (8.0–13.3)	1.00
SOFA score, day-3	11.0 (7.0–12.3)	10.0 (5.5–13.0)	0.66
SOFA score, day-7	6.5 (3.8–11.0)	7.0 (3.0–12.0)	0.91
**Comorbidities**			
Diabetes mellitus	5 (27.8%)	10 (55.6%)	0.18
Congestive heart failure	1 (5.6%)	1 (5.6%)	1.00
COPD	2 (11.1%)	1 (5.6%)	0.50
End-stage renal disease	4 (22.2%)	1 (5.6%)	0.34
Cerebral vascular disease	0 (0%)	4 (22.2%)	0.10
**Laboratory data**			
White blood cell counts (/ml)	11,445.0 (7640.0–13077.5)	11,175.0 (9342.5–12872.5)	0.89
Platelet count (1000/ml)	41.0 (16.5–145.3)	77.5 (44.5–150.0)	0.12
Creatinine (mg/dl)	1.3 (0.8–4.7)	2.1 (0.8–3.1)	0.91
Albumin (mg/dl)	2.8 (2.5–3.2)	3.1 (2.9–3.4)	0.14
Lactate (mg/dl)	29.0 (19.4–51.0)	39.6 (21.1–73.6)	0.30
C-reactive protein (mg/dl)	11.7 (5.1–26.9)	8.2 (3.4–21.1)	0.56
**Outcome**			
Ventilator-day, days	12.0 (8.8–25.3)	12.5 (7.8–18.0)	0.54
ICU-stay, days	14.0 (9.5–25.3)	14.5 (11.8–27.5)	0.74
Hospital day	33.5 (14.8–47.8)	28.5 (22.8–41.3)	0.96
Mortality	8 (44.4%)	2 (11.1%)	0.03

*Data were presented as median (interquartile range) and number (percentage). APACHE II, acute physiology and chronic health evaluation II; SOFA, sequential organ failure assessment; COPD, chronic obstructive pulmonary disease; ICU, intensive care unit*.

### Distinct Dynamic Transcriptome in Immunocompetent and Immunocompromised Patients With Sepsis

**Figure 1:**
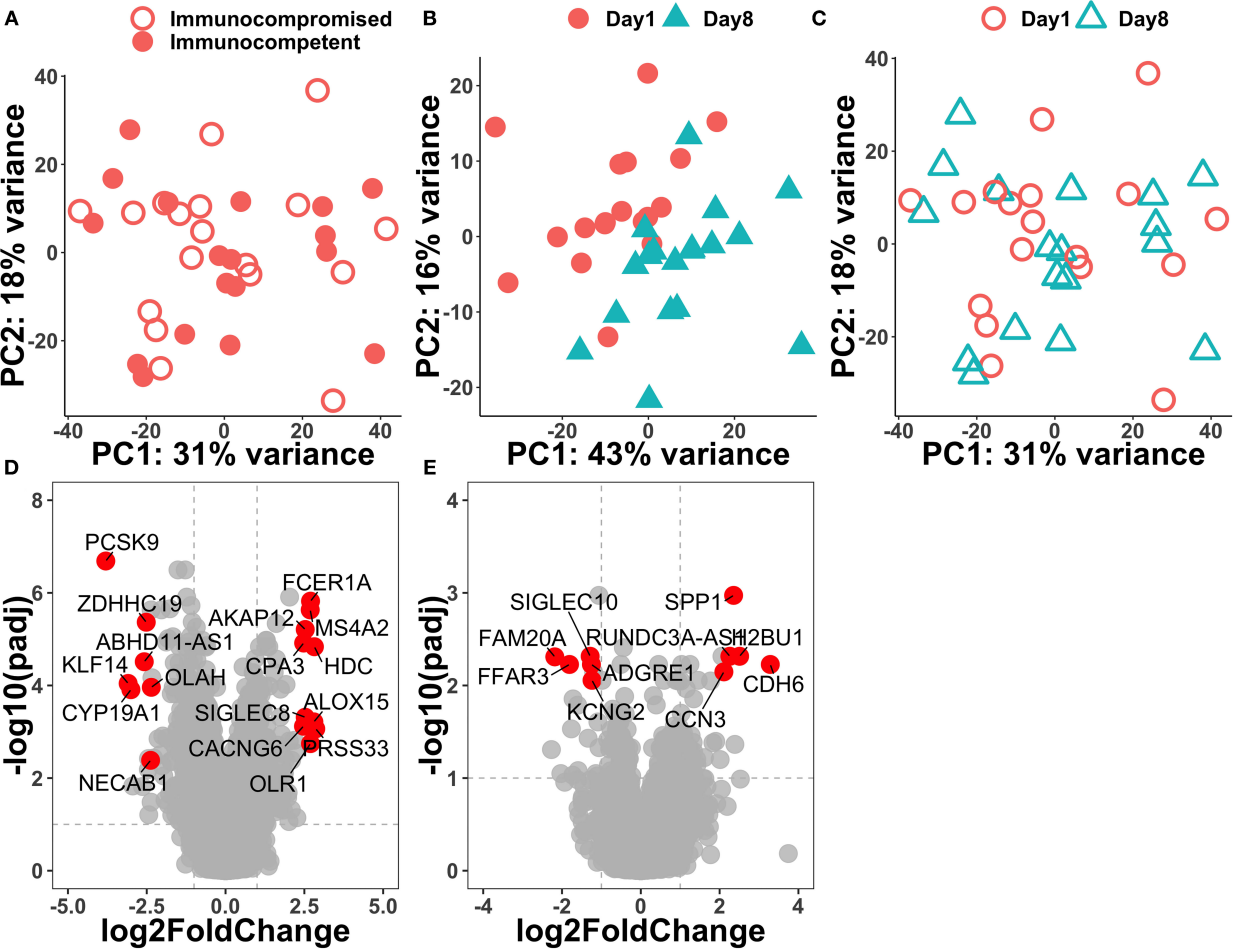
Principal component analysis of day-1 transcriptome among all of the enrolled patients with septic **(A)** and volcano plots of differentially expressed genes between day-1 and day-8 in immunocompetent **(B,D)** and immunocompromised **(C,E)** patients with sepsis.

Principal component analysis was performed on the top 500 variable genes across samples, and we found that the transcriptome was similar on day-1 among immunocompromised and immunocompetent patients (Figure 1A). We found that the separation between day-1 and day-8 was apparently better in the immunocompetent group than those in the immunocompromised group, suggesting the regulated recovery from sepsis in the immunocompetent group (Figures 1B,C). We then compared gene-expression profiles between day-8 and day-1 in the immunocompromised and immunocompetent groups through using the criteria with *p* < 0.01 and log fold change >0.25 or < -0.25 to define differential expression genes (DEGs). In the immunocompetent group, there were 978 DEGs (362 upregulated and 616 downregulated genes), whereas merely 368 DEGs (200 upregulated and 168 downregulated genes) in the immunocompromised group. We found that the top upregulated genes in the immunocompetent group were mainly innate immunity and inflammation relevant genes, namely, *PRSS33, HDC, ALOX15, FCER1A*, and *OLR1*, whereas a significant downregulation was found in *PCSK9*, which has been implicated with increased clearance of endogenous lipids and bacteria among patients with septic with day-3 functional improvement after resuscitation (6) (Figure 1D). In contrast with the highly dynamic transcriptome in immunocompetent patients with septic, we found a relatively blunted dynamic transcriptome among immunocompromised patients with septic (Figure 1E).

### Enriched Pathway of the Dynamic Transcriptome in Immunocompetent Patients With Sepsis

**Figure 2:**
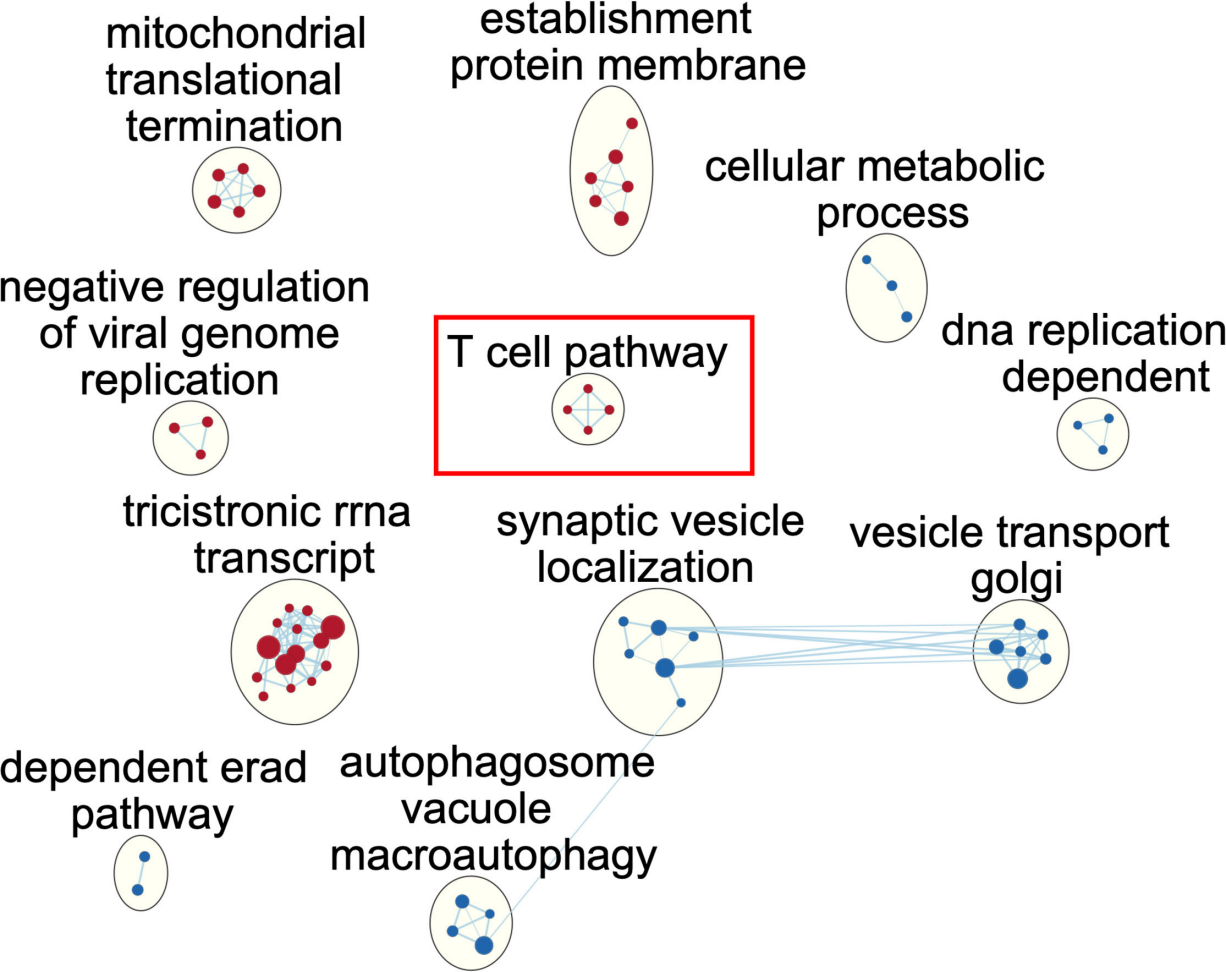
Network of enriched gene ontology term clusters in immunocompetent patients with sepsis. Red circles represent the upregulated pathways, and blue circles represent the downregulated pathways.

To illustrate the alteration of biological pathways in the immunocompetent group, we used GSEA and a functional map to illustrate the enriched pathway. In brief, the identified gene ontology (GO) terms were mapped as a network of gene sets (nodes) related by mutual overlap (edges), and the red and blue nodes represent up and downregulated pathways, respectively. We identified a total of 11 clusters with significant over-represented GO terms using normalised *p* < 0.005 (Figure 2; Supplemental Table 2). We noted that ribosome RNA (rRNA) relevant pathways, such as rRNA biogenesis and metabolic process, were activated, indicating the potential cellular proliferation and tissue repair among immunocompetent patients with septic in the recovery from sepsis. In contrast, autophagy-related pathways, such as macroautophagy, autophagosome organisation, and vesicle budding from the membrane, were downregulated among immunocompetent patients with septic, implicating the resolution of inflammation result from sepsis. Notably, we identified the enriched upregulation of T cell-related pathways, such as T cell selection, positive alpha/beta T cell lineage commitment, and T cell lineage commitment, and the finding indicated the functional T cell immunity in immunocompetent patients was recovered from sepsis. We further used the Reactome to validate our finding in T cell-associated pathway and found an enriched interferon-gamma signalling pathway in which the leading edge genes consisted of *HLA-DRB1, HLA-DPB1, HLA-DPA1, HLA-DRB5, HLA-DQB1*, and *HLA-DRA* (Supplemental Table 3). Collectively, the functional pathway analyses identified the upregulated biogenesis and functional T cell signalling in immunocompetent patients recovered from sepsis.

### Enriched Pathway of the Dynamic Transcriptome in Immunocompromised Patients With Sepsis

**Figure 3:**
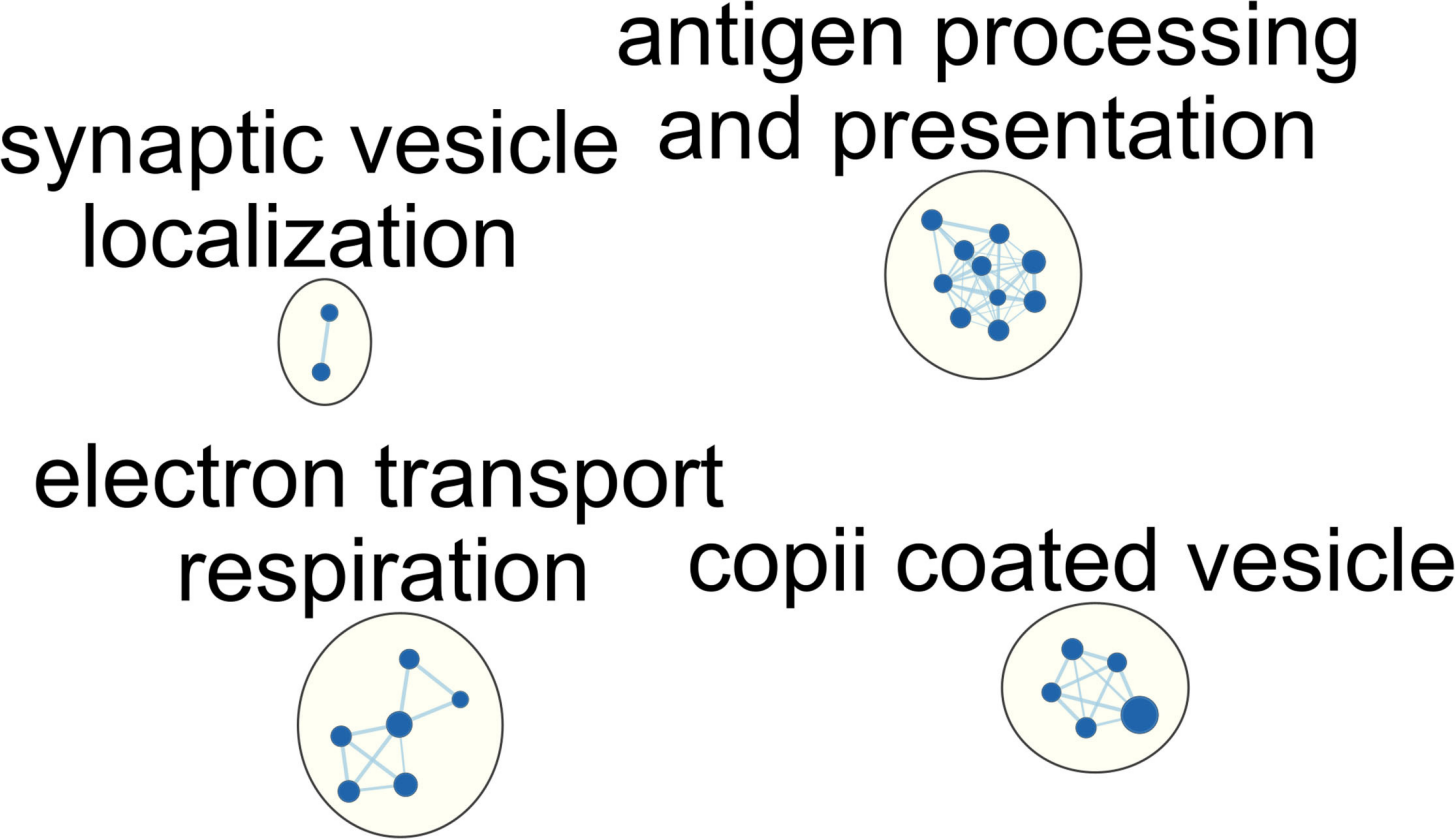
Network of enriched gene ontology term clusters in immunocompromised patients with sepsis. Blue circles represent the downregulated pathways.

We then explored the alteration of the pathway in immunocompromised patients with septic. Similar to the aforementioned limited differentially expressed genes, we identified merely four downregulated pathways, and no upregulated pathway can be enriched. In contrast with the aforementioned upregulated T cell and metabolic process, we found downregulated antigen processing, antigen presentation, and metabolism-associated pathways in immunocompromised patients clinically recovered from sepsis on day-8 (Figure 3; Supplemental Tables 4, 5). In detail, the enriched downregulated metabolism-associated pathways included the tricarboxylic acid (TCA) cycle, aerobic respiration, respiratory electron transport chain, and cellular respiration pathway. These results showed an aberrantly blunted immunological pathways and impaired metabolism status among immunocompromised patients clinically recovered from sepsis.

### MiXCR to Assess the Diversity of TCR

**Figure 4:**
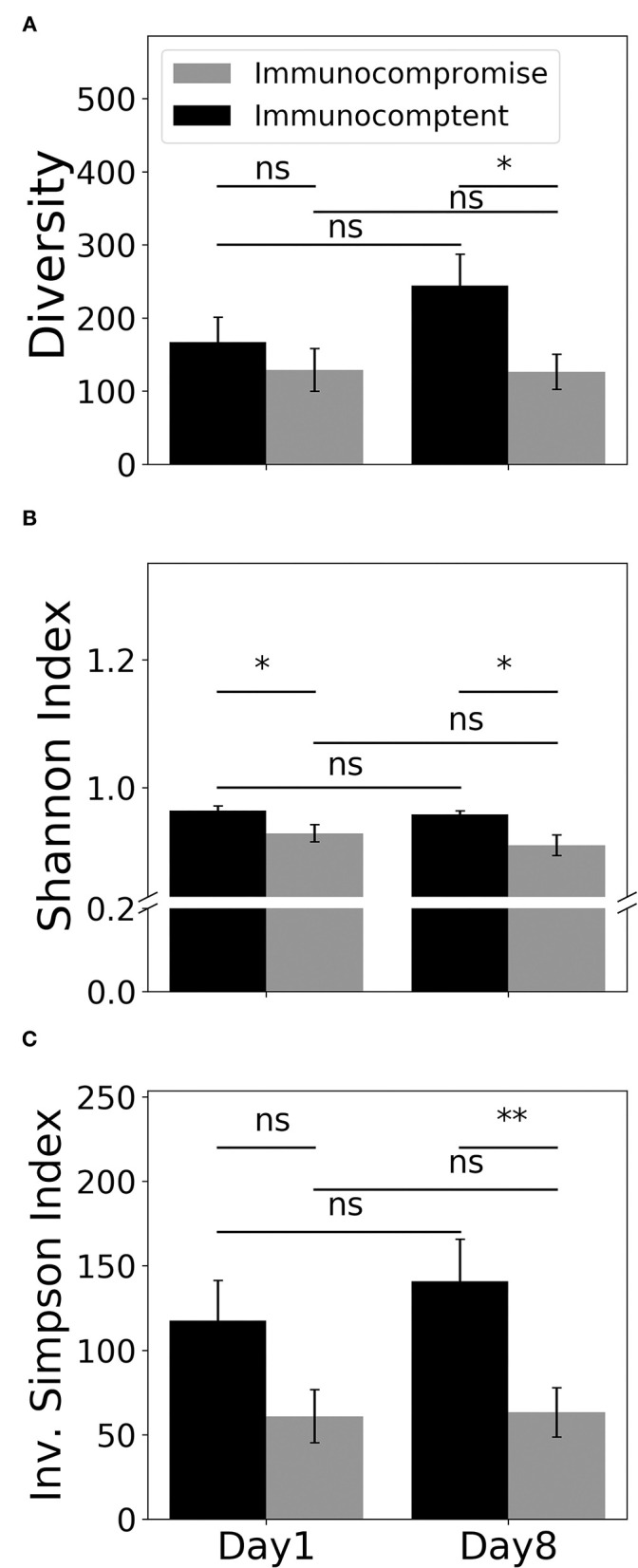
Diversity of the T cell receptor. Counts **(A)**, Shannon's diversity index **(B)**, and inverse Simpson index **(C)** of unique CDR3. (CDR3, complementarity-determining region-3). * < 0.05, ** < 0.005.

Given that we found the distinct T cell-associated pathway between immunocompetent and immunocompromised patients recovered from sepsis, we then employed MiXCR to determine the diversity of TCR through using the number of unique CDR3, Shannon's diversity index, and inverse Simpson index (Figure 4). We found the day-1 number of unique CDR3 tended to be higher in immunocompetent patients than that in immunocompromised patients. Unlike the mildly increased number of unique CDR3 on day-8 among immunocompetent patients, the number of unique CDR3 in immunocompromised patients remained low (Figure 4A). We then used Shannon's diversity index to quantify the diversity of unique CDR3 and found a significantly higher diversity of TCR in immunocompetent patients both on day-1 and on day-8 (Figure 4B). We also used the inverse Simpson index, which reflects abundant clonotypes to assess the diversity of unique CDR3 and found a similar trend with a significantly decreased diversity of TCR in immunocompromised, particularly on day-8 (Figure 4C).

## Discussion

In the present study, we used RNA-Seq, analysed by functional pathway analyses and MiXCR, to identify immunological and metabolic features in immunocompromised patients with sepsis. We found impaired T cell-associated signalling, altered metabolic signalling, and decreased diversity of TCR among immunocompromised patients with septic. The aforementioned immunological and metabolic features indicate a dysregulated recovery in patients with septic with immunocompromised conditions, such as solid cancer underwent therapy, active haematological cancer, and receiving immunosuppressants, due to autoimmune disease or renal transplant recipient. These findings demonstrate the application of RNA-Seq to characterise the subendotype with immunoparalysis in patients with septic, particularly among immunocompromised patients.

The WHO recognises sepsis as a global health priority in 2017 (21), and it is estimated that that sepsis affects 48.9 million patients (38.9–62.9) worldwide and accounts for 19.7% (18.2–21.4) of all global deaths in 2017 (22). Immunocompromised conditions have been found to affect the outcome of sepsis substantially; however, the underlying mechanism remains elusive due to the complexity and high heterogeneity of sepsis (23, 24). The RNA-Seq has been increasingly used to address complex diseases, namely, sepsis, which disrupts the pathophysiological homeostasis result from complex, numerous, intertwined, and subcellular biological events (25). Several studies have used RNA-Seq to identify the subendotype in sepsis, and the identification of subendotype should be essential for individualised medicine in patients with sepsis (26). Antcliffe et al. recently conducted a *post-hoc* transcriptomic analysis among 176 patients in VANISH trial which aimed to explore the mortality impact of vasopressors and corticosteroid in patients with a septic shock and reported two transcriptomic sepsis response signatures (SRSs), such as relatively immunosuppressed signature (SRS-1) and relatively immunocompetent signature (SRS-2) (9). Notably, Antcliffe et al. found that the use of corticosteroids was associated with increased 28-day mortality in patients with septic with SRS-2, whereas the mortality was independent of the use of corticosteroids in patients with septic with SRS-1 (9).

In the present study, the upregulated T cell and Human Leukocyte Antigen (HLA)-associated pathway in immunocompetent patients with septic should be consistent with the aforementioned SRS-2 (Figure 2). The potentially harmful effect of corticosteroid among patients with septic with specific endotype was recently corroborated using the same dataset by Wong et al., whose team has developed a 100-gene-expression array, which was designed to assess adaptive immunity and glucocorticoid receptor signalling in paediatric septic shock (27). In brief, Wong et al. found that among the 44 patients with endotype A, a phenotype with relatively suppressed adaptive immunity, the mortality tended to be higher among the 26 patients receiving corticosteroid compared with 18 patients without corticosteroid (12/26, 46% vs. 4/18, 22%, *p* = 0.105) (27). Accordingly, the aforementioned evidence and our data demonstrated that the transcriptomic signature should have a crucial role in precision medicine among patients with sepsis.

Sepsis is a complex disease with a robust innate response and dysregulated inflammation, which in turn leads to organ dysfunction (28). In the present study, we used over-representation analysis (ORA) to identify several DEGs in immunocompetent patients, and the identified DEGs, namely, *PRSS33, HDC, ALOX15, FCER1A, OLR1*, and *PCSK9*, were mainly innate immunity and cellular damage-associated genes (7, 29–33). For example, *ALOX15* and *FCER1A* were monocyte-associated genes, which have been found to play a pivotal role in the pathogenesis of bacterial sepsis (7, 31). Moreover, we also used CIBERSORT to estimate cellular types and found the relative abundance of monocyte in enrolled patients, and this finding was in line with the increasing data to show the crucial role of monocyte in the pathogenesis of sepsis (Supplemental Figure 1) (7, 31). Notably, the transcriptome was similar on day-1 among immunocompromised and immunocompetent patients (Figure 1A), and the findings highlight the crucial need of using paired day-1 and day-8 samples to address the dynamic transcriptome in patients with septic with high heterogeneity as shown in this study and the previous study (6). Furthermore, we employed functional pathway analysis to demonstrate the essential role of T cell immunity-relevant pathways among immunocompromised patients with sepsis. The aforementioned findings highlight that ORA-based analysis alone may not be adequate to address the complex disease with a high number of differentially expressed genes given that the top DEGs may represent the robust dysregulated immunological and cellular function (34). In contrast, functional pathway-based analyses enable us to identify the distinct T cell-associated pathways as we have shown in the present study (Figure 2).

Several transcriptomic studies have been used to address the T cell immunity in sepsis, and distinct terms were used, such as T cell exhaustion, immunoparalysis, and immune-suppressive signatures (4, 9, 26). Cheng et al. reported an *in vitro* model of innate immunotolerance/immunoparalysis defined by diminished production of lactate and pro-inflammatory cytokines in lipopolysaccharide (LPS) or *Candida albicans*-treated monocytes isolated from patients with sepsis and found not only compromised innate immunity but also impaired energy metabolism in patients with septic with immunoparalysis (4, 26). Increasing evidence has further validated the key role of metabolic abnormality in sepsis (35, 36), and our data also found impaired metabolic pathways, such as TCA cycle and cellular respiration, in immunocompromised patients with sepsis (Figure 3). Additionally, alteration in the metabolic state of T cells has been implicated with the impaired capacity of T cell expansion and effector function of T cells (37, 38). The impaired metabolic signalling as we have shown in this clinical study may hence be associated with the T cell paralysis in the immunocompromised patient with septic, although more mechanistic studies are warranted to elucidate the complex immune-metabolism interaction (4).

Increasing studies have shown the role of T cell exhaustion in patients with sepsis; however, few studies addressed the T cell diversity in patients with septic, particularly immunocompromised patients. Venet et al. conducted a pilot study to explore day-1 and day-7 diversity of TCR in 41 patients with a septic shock through using genomic DNA and multiplex PCR (39). Venet et al. reported that a reduced day-7 TCR β-chain diversity tended to be associated with an increased risk of mortality in the univariable analysis (39). Furthermore, patients who developed a nosocomial infection after the sepsis episode appeared to have a lower day-1 TCR β-chain diversity compared with those without nosocomial infection (39). Recently, there are crucial advances, such as 5′ rapid amplification of cDNA ends (RACE) and measurement of TCR in RNA-level (40). Genomic DNA was widely used to measure the diversity of TCR due to high stability and fixed copy number per cell; however, PCR-associated error result from unused segments and introns remains a major limitation of the DNA-based approach for TCR repertoire (40). In contrast, using RNA with a simplified PCR amplification strategy enables the comprehensive identification of TCR variants and is increasingly applicated (41). MiXCR is a framework that allows for analysing raw sequences of RNA-Seq to determine clonotypes of TCR. Therefore, the approach with MiXCR enables us to simultaneously conduct whole transcriptome and diversity of TCR (11). Similar to our approach, Zhigalova et al. recently demonstrated the utility of using MiXCR and RNA-Seq data to determine the dynamic diversity of TCR among tumour-infiltrating T cells treated with programmed death receptor 1 (PD-1) antibody in mouse melanoma model (42). In the present study, we used RNA-Seq to identify the impaired T cell signalling and MiXCR to reveal the decreased diversity of TCR among immunocompromised patients with septic, and the consistent findings indicate the immunocompromised condition-associated T cell paralysis in sepsis. Similar to our results in immunocompromised patients with septic, Cabrera-Perez et al., conducting qualitative and quantitative analyses of Ag-specific CD4 T cell populations in cecal ligation puncture-treated mice, found a prolonged alteration of Ag-specific T cell repertoire after sepsis despite the recovered number of T cells (43). Collectively, this evidence highlights the feasibility of using data of RNA-Seq and MiXCR to address the diversity of TCR in patients with sepsis and point out the crucial role of reduced diversity of TCR in immunocompromised patients with sepsis.

There are limitations to this study. First, the relatively small number of immunocompromised patients with septic. However, we enrolled severity-matched controls, and the highly comparable controls should allow us the identification of distinct pathways between the two groups. Second, the use of bulk RNA-Seq and future sc-RNA-Seq and functional experiments are warranted for validation at the cellular level. Third, we used RNA-Seq data to assess the diversity of TCR, and future T cell repertoire using 5′RACE should be needed to clarify the issue of TCR in patients with sepsis.

## Conclusion

The increased number of immunocompromised patients with sepsis is a growing issue worldwide, and the RNA-Seq can be used to address the complex immunologic and metabolic features. In the present study, we enrolled immunocompromised patients with septic with severity-matched controls and conducted RNA-Seq with functional pathway and MiXCR analyses. We found that not only dysregulated innate immunity but also compromised T cell function, altered metabolic signalling, and decreased T cell diversity among immunocompromised patients with septic. These data highlight the feasibility of using RNA-Seq to characterise immunologic and metabolic features in immunocompromised patients with septic, and studies using sc-RNA-Seq and 5′RACE are warranted to elucidate the underlying cellular pathway.

## Data Availability Statement

The original contributions presented in the study are publicly available. This data can be found here: https://www.ncbi.nlm.nih.gov/geo/query/acc.cgi?acc=GSE182522.

## Ethics Statement

The studies involving human participants were reviewed and approved by Institutional Review Board approval of the Taichung Veterans General Hospital (CE20069B). The patients/participants provided their written informed consent to participate in this study.

## Author Contributions

P-LC, H-HC, C-YW, C-LW, T-MK, and W-CC: conceived and designed the experiments. P-LC, Y-HJ, and W-CC: acquired data. P-LC, T-HH, and W-CC: contributed materials/analysis tools. P-LC and W-CC: wrote the manuscript. All authors contributed to the article and approved the submitted version.

## Funding

This study was supported in part by grants from Taichung Veterans General Hospital (TCVGH-1104402D, TCVGH-1104403D, and TCVGH-1114401D) and the Taiwanese Ministry of Science and Technology (MOST 109-2314-B-075A-005). The funders had no role in the study design, data collection and analysis, decision to publish or preparation of the manuscript.

## Conflict of Interest

The authors declare that the research was conducted in the absence of any commercial or financial relationships that could be construed as a potential conflict of interest.

## Publisher's Note

All claims expressed in this article are solely those of the authors and do not necessarily represent those of their affiliated organizations, or those of the publisher, the editors and the reviewers. Any product that may be evaluated in this article, or claim that may be made by its manufacturer, is not guaranteed or endorsed by the publisher.
