# Supplementary material for: Using RNA-Seq to Investigate Immune-Metabolism Features in Immunocompromised Patients With Sepsis
Source: Front Med (Lausanne). 2021 Dec 17;8:747263. doi: 10.3389/fmed.2021.747263 (PMC8718501; doi:10.3389/fmed.2021.747263)
Supplement: Supplementary file 1 [file Data_Sheet_1.PDF]

Supplemental table 1. Infection sites and pathogens of the enrolled immunocompromised and severity-matched immunocompetent critically ill septic patients

	**Immunocompromised n=18**	**Immunocompetent n=18**
**Infection cites**		
Pneumonia	12	9
Intra-abdomen infection	2	7
Central nervous system infection	2	0
Urosepsis	1	0
Infective endocarditis	1	0
Skin and soft tissue infection	0	2
**Identified pathogens**		
**Viral infection**		
Influenza[a]	3	1
Rhinovirus/enterovirus[b]	1	0
**Bacterial infection**		
Gram's negative bacilli		
Escherichia coli	3	1
Klebsiella pneumoniae	1	5
Pseudomonas aeruginosa	0	2
Morganella morganii	0	1
Proteus mirabilis	0	1
Gram's positive cocci		
Streptococcus constellatus	1	0
Staphylococcus aureus	0	1
**Fungal infection**		
Pneumocystis jirovecii pneumonia	1	0
Aspergillus fumigatus[c]	1	0
Aspergillus flavus[d]	1	0
**Negative**	6	6

[a]Confirmed by Taiwan Center for Disease Control using RT-PCR

[b]Diagnosed by BioFire FilmArray Respiratory Panel

[c]Proven invasive pulmonary aspergillosis with histopathological, mycological, and radiological evidence.

[d]Probable invasive pulmonary aspergillosis with mycological and radiological evidence.

Supplementary table 2. gene ontology terms of immunocompetent group

Gene ontology term	NES	p-value
**Tricistroni rrna transcript**		
GOBP_MATURATION_OF_SSU_RRNA	1.7949	<0.001
GOBP_NCRNA_METABOLIC_PROCESS	1.7425	<0.001
GOBP_RIBOSOMAL_LARGE_SUBUNIT_BIOGENESIS	1.9480	<0.001
GOBP_RIBOSOME_BIOGENESIS	1.9111	<0.001
GOBP_RIBONUCLEOPROTEIN_COMPLEX_BIOGENESIS	1.8311	<0.001
GOBP_RIBOSOME_ASSEMBLY	1.8393	<0.001
GOBP_MATURATION_OF_LSU_RRNA	1.7421	<0.001
GOBP_RRNA_METABOLIC_PROCESS	1.9586	<0.001
GOBP_SPLICEOSOMAL_SNRNP_ASSEMBLY	1.7431	<0.001
GOBP_MATURATION_OF_SSU_RRNA_FROM_TRICISTRONIC_RRNA_TRANSCRIPT_SSU_RRNA_5_8S_RRNA_LSU_RRNA	1.8044	<0.001
GOBP_RIBOSOMAL_SMALL_SUBUNIT_BIOGENESIS	1.8825	<0.001
GOBP_NCRNA_PROCESSING	1.8032	<0.001
GOBP_RIBOSOMAL_LARGE_SUBUNIT_ASSEMBLY	1.6880	0.0023
GOBP_CLEAVAGE_INVOLVED_IN_RRNA_PROCESSING	1.7123	0.0024
**Establishment protein membrane**		
GOBP_ESTABLISHMENT_OF_PROTEIN_LOCALIZATION_TO_ENDOPLASMIC_RETICULUM	1.6530	<0.001
GOBP_NUCLEAR_TRANSCRIBED_MRNA_CATABOLIC_PROCESS_NONSENSE_MEDIATED_DECAY	1.9904	<0.001
GOBP_VIRAL_GENE_EXPRESSION	1.8505	<0.001
GOBP_TRANSLATIONAL_INITIATION	1.8307	<0.001
GOBP_COTRANSLATIONAL_PROTEIN_TARGETING_TO_MEMBRANE	1.9644	<0.001
GOBP_CYTOPLASMIC_TRANSLATION	1.7832	<0.001
**Vesicle transport golgi**		
GOBP_VESICLE_TARGETING	-1.8790	<0.001
GOBP_VESICLE_BUDDING_FROM_MEMBRANE	-1.7198	<0.001

GOBP_ENDOPLASMIC_RETICULUM_TO_GOLGI_VESICLE_MEDIATED_TRANSPORT	-1.8080	<0.001
GOBP_GOLGI_VESICLE_TRANSPORT	-1.7415	<0.001
GOBP_COPII_COATED_VESICLE_BUDDING	-1.7711	<0.001
GOBP_VESICLE_TARGETING_TO_FROM_OR_WITHIN_GOLGI	-1.8195	<0.001
**Synaptic vesicle localization**		
GOBP_VESICLE_LOCALIZATION	-1.7748	<0.001
GOBP_SYNAPTIC_VESICLE_LOCALIZATION	-1.6954	<0.001
GOBP_SYNAPTIC_VESICLE_TRANSPORT	-1.6648	<0.001
GOBP_VESICLE_ORGANIZATION	-1.7467	<0.001
GOBP_CELLULAR_PIGMENTATION	-1.6737	0.0017
GOBP_MULTIVESICULAR_BODY_ORGANIZATION	-1.6780	0.0035
**Mitochondria translational termination**		
GOBP_MITOCHONDRIAL_GENE_EXPRESSION	1.8858	<0.001
GOBP_TRANSLATIONAL_ELONGATION	1.7497	<0.001
GOBP_MITOCHONDRIAL_TRANSLATIONAL_TERMINATION	1.8478	<0.001
GOBP_MITOCHONDRIAL_TRANSLATION	1.9349	<0.001
GOBP_TRANSLATIONAL_TERMINATION	1.8665	<0.001
**Autophagosome vacuole macroautophagy**		
GOBP_MACROAUTOPHAGY	-1.6639	<0.001
GOBP_AUTOPHAGOSOME_ORGANIZATION	-1.7563	<0.001
GOBP_VACUOLE_ORGANIZATION	-1.7939	<0.001
GOBP_REGULATION_OF_AUTOPHAGOSOME_ASSEMBLY	-1.6848	0.0018
**T cell pathway**		
GOBP_POSITIVE_T_CELL_SELECTION	1.7710	<0.001
GOBP_T_CELL_SELECTION	1.8011	<0.001
GOBP_T_CELL_LINEAGE_COMMITMENT	1.7123	<0.001
GOBP_CD4_POSITIVE_OR_CD8_POSITIVE_ALPHA_BETA_T_CELL_LINEAGE_COMMITMENT	1.7134	0.0022

**Dna replication depedent**		
GOBP_RDNA_HETEROCHROMATIN_ASSEMBLY	-1.7289	<0.001
GOBP_DNA_REPLICATION_DEPENDENT_NUCLEOSOME_ORGANIZATION	-1.6922	<0.001
GOBP_NEGATIVE_REGULATION_OF_MEGAKARYOCYTE_DIFFERENTIATION	-1.6855	<0.001
**Cellular metabolic process**		
GOBP_POLYSACCHARIDE_CATABOLIC_PROCESS	-1.6999	<0.001
GOBP_CELLULAR_GLUCAN_METABOLIC_PROCESS	-1.7013	<0.001
GOBP_CELLULAR_CARBOHYDRATE_BIOSYNTHETIC_PROCESS	-1.6544	<0.001
**Negative regulation of viral genome replication**		
GOBP_REGULATION_OF_VIRAL_GENOME_REPLICATION	1.6923	<0.001
GOBP_NEGATIVE_REGULATION_OF_VIRAL_PROCESS	1.6789	<0.001
GOBP_NEGATIVE_REGULATION_OF_VIRAL_GENOME_REPLICATION	1.7218	0.0024
**Dependent erad pathway**		
GOBP_UBIQUITIN_DEPENDENT_ERAD_PATHWAY	-1.7513	<0.001
GOBP_ERAD_PATHWAY	-1.6690	<0.001

Spplementary table 3. REACTOME terms of immunocompetent group

REACTOME term	NES	p-value
**Senescence expression dna**		
REACTOME_CELLULAR_SENESCENCE	-1.5840	<0.001
REACTOME_PRE_NOTCH_EXPRESSION_AND_PROCESSING	-1.7938	<0.001
REACTOME_HDACS_DEACETYLATE_HISTONES	-1.7647	<0.001
REACTOME_ACTIVATED_PKN1_STIMULATES_TRANSCRIPTION_OF_AR_ANDROGEN_RECEPTOR_REGULATED_GENES_KLK2_AND_KLK3	-1.8983	<0.001
REACTOME_AMYLOID_FIBER_FORMATION	-1.6348	<0.001
REACTOME_ESTROGEN_DEPENDENT_GENE_EXPRESSION	-1.6261	<0.001
REACTOME_NONHOMOLOGOUS_END_JOINING_NHEJ	-1.6801	<0.001
REACTOME_HATS_ACETYLATE_HISTONES	-1.6513	<0.001
REACTOME_REPRODUCTION	-1.568	<0.001
REACTOME_MEIOTIC_SYNAPSIS	-1.8085	<0.001
REACTOME_CONDENSATION_OF_PROPHASE_CHROMOSOMES	-1.7330	<0.001
REACTOME_MEIOSIS	-1.5874	<0.001
REACTOME_HDMS_DEMETHYLATE_HISTONES	-1.7868	<0.001
REACTOME_RECOGNITION_AND_ASSOCIATION_OF_DNA_GLYCOSYLASE_WITH_SITE_CONTAINING_AN_AFFECTED_PURINE	-1.7738	<0.001
REACTOME_ESR_MEDIATED_SIGNALING	-1.6228	<0.001
REACTOME_INHIBITION_OF_DNA_RECOMBINATION_AT_TELOMERE	-1.7613	<0.001
REACTOME_SIRT1_NEGATIVELY_REGULATES_RRNA_EXPRESSION	-1.8480	<0.001
REACTOME_HCMV_LATE_EVENTS	-1.7036	<0.001
REACTOME_MEIOTIC_RECOMBINATION	-1.6146	<0.001
REACTOME_OXIDATIVE_STRESS_INDUCED_SENESCENCE	-1.6529	<0.001
REACTOME_ERCC6_CSB_AND_EHMT2_G9A_POSITIVELY_REGULATE_RRNA_EXPRESSION	-1.6552	<0.001

REACTOME_MITOTIC_PROPHASE	-1.5399	<0.001
REACTOME_SENESCENCE_ASSOCIATED_SECRETORY_PHENOTYPE_SASP	-1.7167	<0.001
REACTOME_PRC2_METHYLATES_HISTONES_AND_DNA	-1.6707	<0.001
REACTOME_RHO_GTPASES_ACTIVATE_PKNS	-1.8439	<0.001
REACTOME_NEGATIVE_EPIGENETIC_REGULATION_OF_RRNA_EXPRESSION	-1.5460	<0.001
REACTOME_DNA_METHYLATION	-1.7618	<0.001
REACTOME_RUNX1_REGULATES_GENES_INVOLVED_IN_MEGAKARYOCYTE_DIFFERENTIATION_AND_PLATELET_FUNCTION	-1.6640	<0.001
REACTOME_DNA_DAMAGE_TELOMERE_STRESS_INDUCED_SENESCENCE	-1.7467	0.0016
REACTOME_RNA_POLYMERASE_I_PROMOTER_ESCAPE	-1.6658	0.0016
REACTOME_TRANSCRIPTIONAL_REGULATION_OF_GRANULOPOIESIS	-1.5728	0.0016
REACTOME_B_WICH_COMPLEX_POSITIVELY_REGULATES_RRNA_EXPRESSION	-1.5563	0.0017
REACTOME_BASE_EXCISION_REPAIR_AP_SITE_FORMATION	-1.6820	0.005
**Mitochondrial translation initiation**		
REACTOME_RRNA_PROCESSING	2.2214	<0.001
REACTOME_RESPONSE_OF_EIF2AK4_GCN2_TO_AMINO_ACID_DEFICIENCY	2.1185	<0.001
REACTOME_SELENOAMINO_ACID_METABOLISM	2.0603	<0.001
REACTOME_MITOCHONDRIAL_TRANSLATION	1.8541	<0.001
REACTOME_CELLULAR_RESPONSE_TO_STARVATION	1.6324	<0.001
REACTOME_EUKARYOTIC_TRANSLATION_ELONGATION	2.2084	<0.001
REACTOME_ACTIVATION_OF_THE_MRNA_UPON_BINDING_OF_THE_CAP_BINDING_COMPLEX_AND_EIFS_AND_SUBSEQUENT_BINDING_TO_43S	2.0027	<0.001
REACTOME_NONSENSE_MEDIATED_DECAY_NMD	2.0325	<0.001
REACTOME_TRANSLATION	2.0868	<0.001
REACTOME_REGULATION_OF_EXPRESSION_OF_SLITS_AND_ROBOS	1.7865	<0.001
REACTOME_SIGNALING_BY_ROBO_RECEPTORS	1.6079	<0.001

REACTOME_INFLUENZA_INFECTION	1.8652	<0.001
REACTOME_SRP_DEPENDENT_COTRANSLATIONAL_PROTEIN_TARGETING_TO_MEMBRANE	2.0170	<0.001
REACTOME_EUKARYOTIC_TRANSLATION_INITIATION	2.1922	<0.001
REACTOME_RRNA_MODIFICATION_IN_THE_NUCLEUS_AND_CYTOSOL	1.9427	<0.001
**Transport golgi er**		
REACTOME_TRANSPORT_TO_THE_GOLGI_AND_SUBSEQUENT_MODIFICATION	-1.7634	<0.001
REACTOME_ER_TO_GOLGI_ANTEROGRADE_TRANSPORT	-1.7797	<0.001
REACTOME_INTRA_GOLGI_AND_RETROGRADE_GOLGI_TO_ER_TRAFFIC	-1.5453	<0.001
REACTOME_COPII_MEDIATED_VESICLE_TRANSPORT	-1.7643	<0.001
REACTOME_COPI_MEDIATED_ANTEROGRADE_TRANSPORT	-1.6569	<0.001
REACTOME_CARGO_CONCENTRATION_IN_THE_ER	-1.6927	0.0018
**Immunoregulatory interactions lymphoid**		
REACTOME_CD22_MEDIATED_BCR_REGULATION	1.7373	<0.001
REACTOME_IMMUNOREGULATORY_INTERACTIONS_BETWEEN_A_LYMPHOID_AND_A_NON_LYMPHOID_CELL	1.8764	<0.001
REACTOME_INITIAL_TRIGGERING_OF_COMPLEMENT	1.8579	<0.001
REACTOME_CREATION_OF_C4_AND_C2_ACTIVATORS	1.9154	<0.001
REACTOME_BINDING_AND_UPTAKE_OF_LIGANDS_BY_SCAVENGER_RECEPTORS	1.6840	<0.001
**Irak4 deficiency tlr2**		
REACTOME_DISEASES_OF_IMMUNE_SYSTEM	-1.6705	<0.001
REACTOME_REGULATION_OF_TLR_BY_ENDOGENOUS_LIGAND	-1.6407	<0.001
REACTOME_TOLL_LIKE_RECEPTOR_CASCADES	-1.5444	<0.001
REACTOME_IRAK4_DEFICIENCY_TLR2_4	-1.6119	0.0019
**Pd1 signaling**		
REACTOME_PD_1_SIGNALING	1.8598	<0.001

REACTOME_GENERATION_OF_SECOND_MESSENGER_MOLECULES	1.8280	<0.001
REACTOME_COSTIMULATION_BY_THE_CD28_FAMILY	1.6385	0.0024
**Interferon gamma signaling**		
REACTOME_INTERFERON_SIGNALING	1.6024	<0.001
REACTOME_INTERFERON_GAMMA_SIGNALING	1.6161	<0.001
REACTOME_INTERFERON_ALPHA_BETA_SIGNALING	1.5999	<0.001
**Rab regulation trafficking**		
REACTOME_RAB_GEFS_EXCHANGE_GTP_FOR_GDP_ON_RABS	-1.6390	<0.001
REACTOME_RAB_REGULATION_OF_TRAFFICKING	-1.6361	<0.001
REACTOME_RAB_GERANYLGERANYLATION	-1.626	<0.001
**Activation pre complex**		
REACTOME_ACTIVATION_OF_THE_PRE_REPLICATIVE_COMPLEX	1.7929	<0.001
REACTOME_ACTIVATION_OF_ATR_IN_RESPONSE_TO_REPLICATION_STRESS	1.6555	0.0047
**Resolution ap sites**		
REACTOME_DNA_STRAND_ELONGATION	1.7592	0.0022
REACTOME_RESOLUTION_OF_AP_SITES_VIA_THE_MULTIPLE_NUCLEOTIDE_PATCH_REPLACEMENT_PATHWAY	1.5549	0.0023

Supplementary table 4. gene ontology terms of Immunocompromised group

Gene ontology term	NES	p-value
**Antigen processing and presentation**		
GOBP_ANTIGEN_PROCESSING_AND_PRESENTATION_OF_PEPTIDE_ANTIGEN_VIA_MHC_CLASS_I	-2.0423	<0.001
GOBP_INNATE_IMMUNE_RESPONSE_ACTIVATING_SIGNAL_TRANSDUCTION	-1.8562	<0.001
GOBP_REGULATION_OF_CELLULAR_AMINE_METABOLIC_PROCESS	-1.98	<0.001
GOBP_INTERLEUKIN_1_MEDIATED_SIGNALING_PATHWAY	-1.8467	<0.001
GOBP_ANTIGEN_PROCESSING_AND_PRESENTATION_OF_EXOGENOUS_PEPTIDE_ANTIGEN_VIA_MHC_CLASS_I	-1.9642	<0.001
GOBP_ACTIVATION_OF_INNATE_IMMUNE_RESPONSE	-1.8472	<0.001
GOBP_PROTEASOMAL_UBIQUITIN_INDEPENDENT_PROTEIN_CATABOLIC_PROCESS	-1.7906	<0.001
GOBP_SCF_DEPENDENT_PROTEASOMAL_UBIQUITIN_DEPENDENT_PROTEIN_CATABOLIC_PROCESS	-1.7118	<0.001
GOBP_REGULATION_OF_CELLULAR_AMINO_ACID_METABOLIC_PROCESS	-1.9973	<0.001
GOBP_ANAPHASE_PROMOTING_COMPLEX_DEPENDENT_CATABOLIC_PROCESS	-1.7402	<0.001
**Electron transport respiration**		
GOBP_RESPIRATORY_ELECTRON_TRANSPORT_CHAIN	-1.7893	<0.001
GOBP_CELLULAR_RESPIRATION	-1.8543	<0.001
GOBP_AEROBIC_RESPIRATION	-1.9122	<0.001
GOBP_ATP_SYNTHESIS_COUPLED_ELECTRON_TRANSPORT	-1.8388	<0.001
GOBP_OXIDATIVE_PHOSPHORYLATION	-1.8436	<0.001
GOBP_TRICARBOXYLIC_ACID_CYCLE	-1.7286	0.0026
**Copii coated vesicle**		
GOBP_VESICLE_TARGETING	-1.758	<0.001
GOBP_VESICLE_BUDDING_FROM_MEMBRANE	-1.7012	<0.001
GOBP_COPII_COATED_VESICLE_BUDDING	-1.7206	<0.001
GOBP_GOLGI_VESICLE_TRANSPORT	-1.7123	<0.001
GOBP_VESICLE_TARGETING_TO_FROM_OR_WITHIN_GOLGI	-1.7537	<0.001
**Synaptic vesicle localization**		

GOBP_SYNAPTIC_VESICLE_LOCALIZATION	·1.7171	<0.001
GOBP_SYNAPTIC_VESICLE_TRANSPORT	·1.8055	0.0026

Supplemental Table 5. REACTOME terms of immunocompromised group

Reactome term	NES	p-value
**beta signalling cell**		
REACTOME_METABOLISM_OF_POLYAMINES	-2.055	<0.001
REACTOME_SIGNALING_BY_NOTCH4	-1.6609	<0.001
REACTOME_ABC_TRANSPORTER_DISORDERS	-1.7521	<0.001
REACTOME_CELLULAR_RESPONSE_TO_HYPOXIA	-1.7625	<0.001
REACTOME_PCP_CE_PATHWAY	-1.7171	<0.001
REACTOME_DECTIN_1_MEDIATED_NONCANONICAL_NF_KB_SIGNALING	-2.007	<0.001
REACTOME_ANTIGEN_PROCESSING_CROSS_PRESENTATION	-1.8931	<0.001
REACTOME_REGULATION_OF_PTEN_STABILITY_AND_ACTIVITY	-2.0098	<0.001
REACTOME_CLEC7A_DECTIN_1_SIGNALING	-1.9916	<0.001
REACTOME_DEFECTIVE_CFTR_CAUSES_CYSTIC_FIBROSIS	-2.0229	<0.001
REACTOME_DECTIN_2_FAMILY	-1.7271	<0.001
REACTOME_REGULATION_OF_MRNA_STABILITY_BY_PROTEINS_THAT_BIND_AU_RICH_ELEMENTS	-1.9673	<0.001
REACTOME_UCH_PROTEINASES	-1.6237	<0.001
REACTOME_INTERLEUKIN_1_SIGNALING	-2.0273	<0.001
REACTOME_CELLULAR_RESPONSE_TO_CHEMICAL_STRESS	-1.7708	<0.001
REACTOME_REGULATION_OF_HMOX1_EXPRESSION_AND_ACTIVITY	-2.0958	<0.001
REACTOME_STABILIZATION_OF_P53	-1.7527	<0.001
REACTOME_REGULATION_OF_RAS_BY_GAPS	-1.6532	<0.001
REACTOME_REGULATION_OF_RUNX3_EXPRESSION_AND_ACTIVITY	-1.8217	<0.001
REACTOME_NEGATIVE_REGULATION_OF_NOTCH4_SIGNALING	-1.9556	<0.001
REACTOME_TNFR2_NON_CANONICAL_NF_KB_PATHWAY	-1.5608	<0.001
REACTOME_CYTOPROTECTION_BY_HMOX1	-1.7106	<0.001
REACTOME_HIV_INFECTION	-1.7053	<0.001

REACTOME_ORC1_REMOVAL_FROM_CHROMATIN	-1.5995	<0.001
REACTOME_APC_C_CDH1_MEDIATED_DEGRADATION_OF_CDC20_AND_OTHER_APC_C_CDH1_TARGETED_PROTEINS_IN_LATE_MITOSIS_EARLY_G1	-1.9248	<0.001
REACTOME_HEDGEHOG_LIGAND_BIOGENESIS	-1.9572	<0.001
REACTOME_DEGRADATION_OF_DVL	-1.8795	<0.001
REACTOME_CLASS_I_MHC_MEDIATED_ANTIGEN_PROCESSING_PRESENTATION	-1.6528	<0.001
REACTOME_HOST_INTERACTIONS_OF_HIV_FACTORS	-1.5796	<0.001
REACTOME_ASYMMETRIC_LOCALIZATION_OF_PCP_PROTEINS	-1.6834	<0.001
REACTOME_DOWNSTREAM_SIGNALING_EVENTS_OF_B_CELL_RECEPTOR_BCR	-1.915	<0.001
REACTOME_INTERLEUKIN_1_FAMILY_SIGNALING	-1.8745	<0.001
REACTOME_APC_C_MEDIATED_DEGRADATION_OF_CELL_CYCLE_PROTEINS	-1.8329	<0.001
REACTOME_CROSS_PRESENTATION_OF_SOLUBLE_EXOGENOUS_ANTIGENS_ENDOSOMES	-1.8739	<0.001
REACTOME_REGULATION_OF_RUNX2_EXPRESSION_AND_ACTIVITY	-1.9534	<0.001
REACTOME_AUF1_HNRNP_D0_BINDS_AND_DESTABILIZES_MRNA	-1.9799	<0.001
REACTOME_HEDGEHOG_ON_STATE	-1.7351	<0.001
REACTOME_DEGRADATION_OF_GLI1_BY_THE_PROTEASOME	-2.008	<0.001
REACTOME_BETA_CATENIN_INDEPENDENT_WNT_SIGNALING	-1.7235	<0.001
REACTOME_DEGRADATION_OF_AXIN	-1.932	<0.001
REACTOME_SIGNALING_BY_THE_B_CELL_RECEPTOR_BCR	-1.7163	<0.001
REACTOME_C_TYPE_LECTIN_RECEPTORS_CLRS	-1.9844	<0.001
REACTOME_MAPK6_MAPK4_SIGNALING	-1.7644	<0.001
REACTOME_SCF_SKP2_MEDIATED_DEGRADATION_OF_P27_P21	-1.8218	0.0029
REACTOME_TRANSCRIPTION_OF_THE_HIV_GENOME	-1.6247	0.0031
REACTOME_ASSEMBLY_OF_THE_PRE_REPLICATIVE_COMPLEX	-1.613	0.0031
REACTOME_DEGRADATION_OF_BETA_CATENIN_BY_THE_DESTRUCTION_COMPLEX	-1.8093	0.0031
REACTOME_G1_S_DNA_DAMAGE_CHECKPOINTS	-1.6339	0.0032

**tca cycle respiratory**		
REACTOME_RESPIRATORY_ELECTRON_TRANSPORT_ATP_SYNTHESIS_BY_CHEMIOSMOTIC_COUPLING_AND_HEAT_PRODUCTION_BY_UNCOUPLING_PROTEINS	-1.6538	<0.001
REACTOME_THE_CITRIC_ACID_TCA_CYCLE_AND_RESPIRATORY_ELECTRON_TRANSPORT	-1.697	<0.001
REACTOME_CITRIC_ACID_CYCLE_TCA_CYCLE	-1.6525	0.0026
REACTOME_CRISTAE_FORMATION	-1.6628	0.0028
**receptor tlr2 cascade**		
REACTOME_IRAK4_DEFICIENCY_TLR2_4	-1.7988	<0.001
REACTOME_DISEASES_OF_IMMUNE_SYSTEM	-1.7337	<0.001
REACTOME_TOLL_LIKE_RECEPTOR_TLR1_TLR2_CASCADE	-1.5597	<0.001
**cargo concentration er**		
REACTOME_COPII_MEDIATED_VESICLE_TRANSPORT	-1.7132	<0.001
REACTOME_CARGO_CONCENTRATION_IN_THE_ER	-1.5917	0.0025

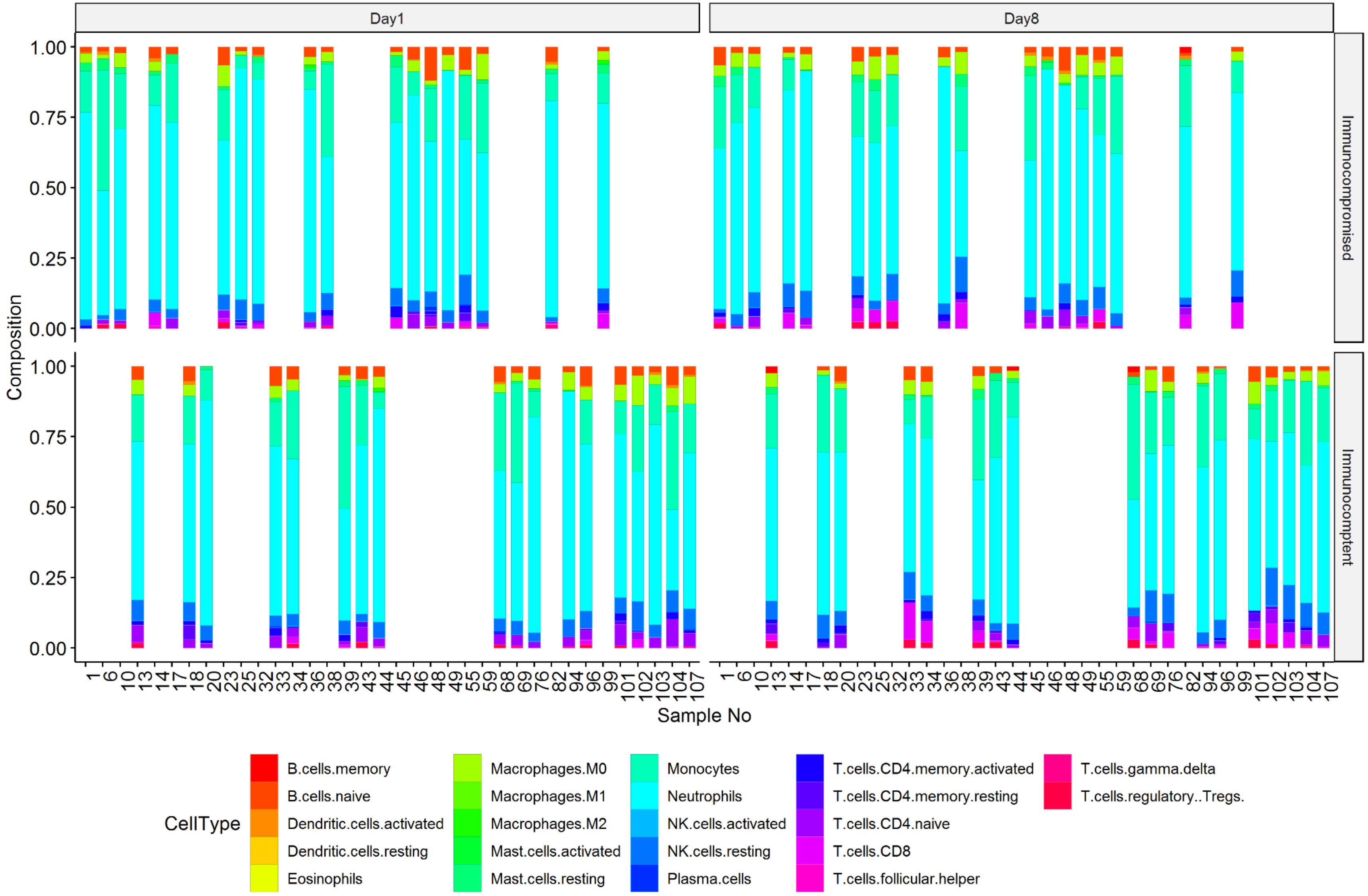

Supplemental figure 1. The use of CIBERSORT to estimate cellular type
